# A case report from non-endemic Australia on systemic melioidosis presenting with septic arthritis

**DOI:** 10.1016/j.imj.2024.100161

**Published:** 2024-12-31

**Authors:** Buddhika Dhananjalee Alahakoon, Monarita Handa, Shiromali Malalasekara

**Affiliations:** Department of Medicine, Rockhampton Base Hospital, Rockhampton 4700, Australia

**Keywords:** Tropical infections, Melioidosis, Non-endemic area, Diagnostic delay, Septic arthritis

## Abstract

Clinical spectrum of melioidosis can vary from a simple skin infection and pneumonia to severe septicaemia with multiorgan failure. Bone involvement in melioidosis is generally low, and the major risk factor is the delay in diagnosing the primary site infection. We present a case of septic arthritis with primary lung melioidosis, whose diagnosis of pulmonary melioidosis was delayed for 5 weeks leading to a septicaemia and septic arthritis. This case highlights the importance of improved clinical awareness among health practitioners and a low threshold for radiological screening of high-risk patients, even in non-endemic areas. It also highlights the fact that having adjunctive open arthrotomy in managing joint infection in melioidosis improves the clinical response to treatment.

## 1 Introduction

*Burkholderia pseudomallei* is a Gram-negative pathogen that is native to the soils of Northern Australia and Southeast Asia. It is the causative agent of melioidosis, a disease that can range in severity from skin infections and pneumonia to disseminated illness with fulminant septicaemia. Melioidosis, is also known as the “great mimicker,” stems from the fact that diagnosis is difficult because it mimics many other disorders, particularly tuberculosis.^1^ Despite improvements in antibiotic therapy, intensive care facilities and better diagnostic modalities, melioidosis still remains a disease associated with a significant mortality attributable to severe sepsis and its complications, with a mortality rate ranging from 6% in Australia to 40% in Southeast Asia.^2^

## 2 Case report

A 57-year-old construction site worker with poorly controlled diabetes mellitus and a history of heavy alcohol consumption presented with a 5-week history of low-grade fever progressive shortness of breath and cough with productive sputum. He has received two courses of oral antibiotics for presumed community acquired pneumonia without any clinical response to treatment. He presented to the emergency department on the 5th week of the illness, complaining of painful swelling of the left elbow. The next day, he developed right ankle joint swelling.

On examination he was febrile with temperature of 101 ℉. He was tachycardic with a pulse rate of 120 bpm and a blood pressure of 100/60 mmHg. There were no audible murmurs. His oxygen saturation on air was 98% with a respiratory rate of 16 breaths per minute. His respiratory examination revealed right apical bronchial breathing patch. Left elbow and right ankle examinations revealed tender, swollen, red and warm joints with effusions.

Basic blood investigations were as follows Table 1.

**Table 1:** Basic investigations.

Investigation	Value	Reference range
White Blood Cells Neutrophils (/L)	11.30 × 10^9^	2.0–8.0
Heamoglobin (g/L)	128	135–180
Platelets (/L)	353 × 10^9^	140–400
CRP (mg/L)	200	<5.0
Random Blood Sugar (mmol/L)	15.5	3.0–7.8
Sodium (mmol/L)	135	135–145
Potassium (mmol/L)	3.6	3.5–5.2
HbA1C (%)	12.4	4.3–6.0
Serum Creatinine (μmol/L)/ Glomerular Filtration Rate [mL/(min⋅1.73m^2^)]	46/>90	60–110/>90
Bilirubin (Total, μmol/L)	14	<20
Bilirubin (Conjugated, μmol/L)	7	<4
Alkaline Phosphatase (nmol/L)	113	30–110
Gamma-GT (units/L)	111	<55
Alanine Transaminase (U/L)	236	<45
Aspartate Transaminase (U/L)	227	<35
Lactate Dehydrogenase (U/L)	516	135–225
Procalcitonin (ng/mL)	0.8	<0.1
Erythrocyte Sedimentation Rate (mm/h)	90	<20

*Abbreviations*: CRP, C-reactive protein; Gamma-GT, gamma glutamyl transferase.

**Fig. 1:**
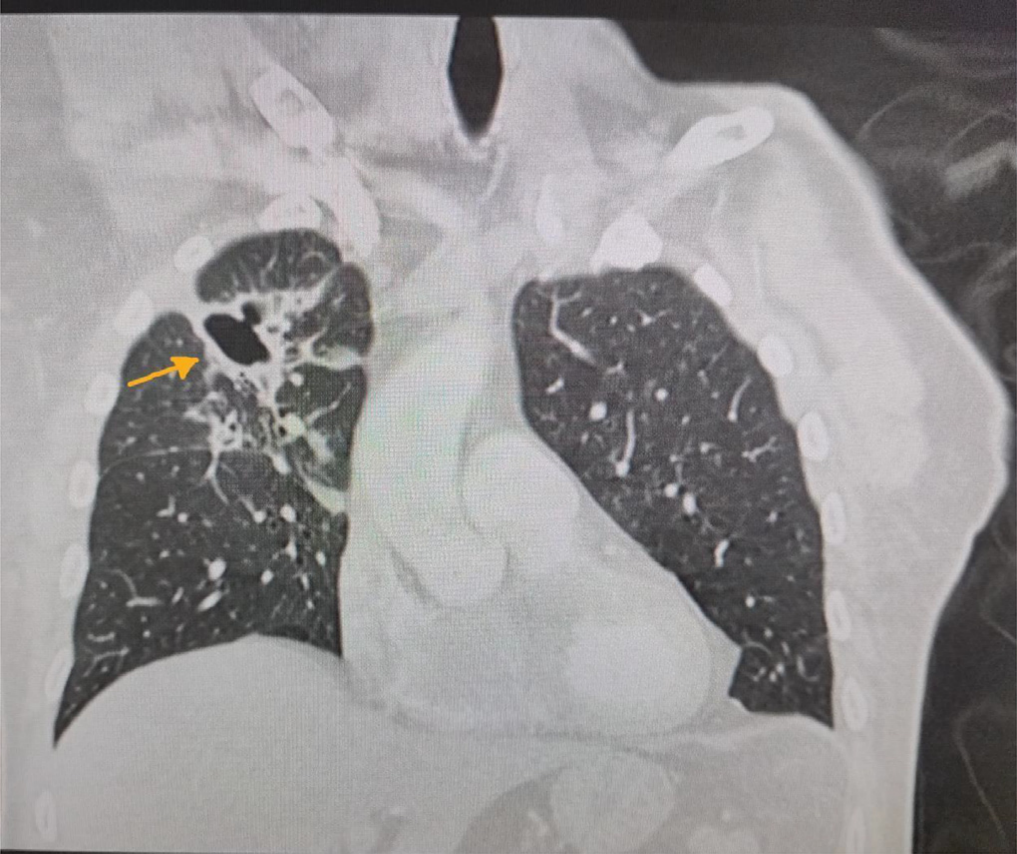
Contrast enhanced computer tomography. Yellow arrow points to cystic cavity with a thick wall and right upper lobe bronchiectasis.

X-ray and Contrast Enhanced Computer Tomography (CECT) chest were reported as “bronchiectasis in the right upper lobe with associated atelectasis and some loss of volume. Largest cystic cavity seems to demonstrate a thick wall. Appearances favouring benign/infective changes” (Fig. 1).

**Fig. 2:**
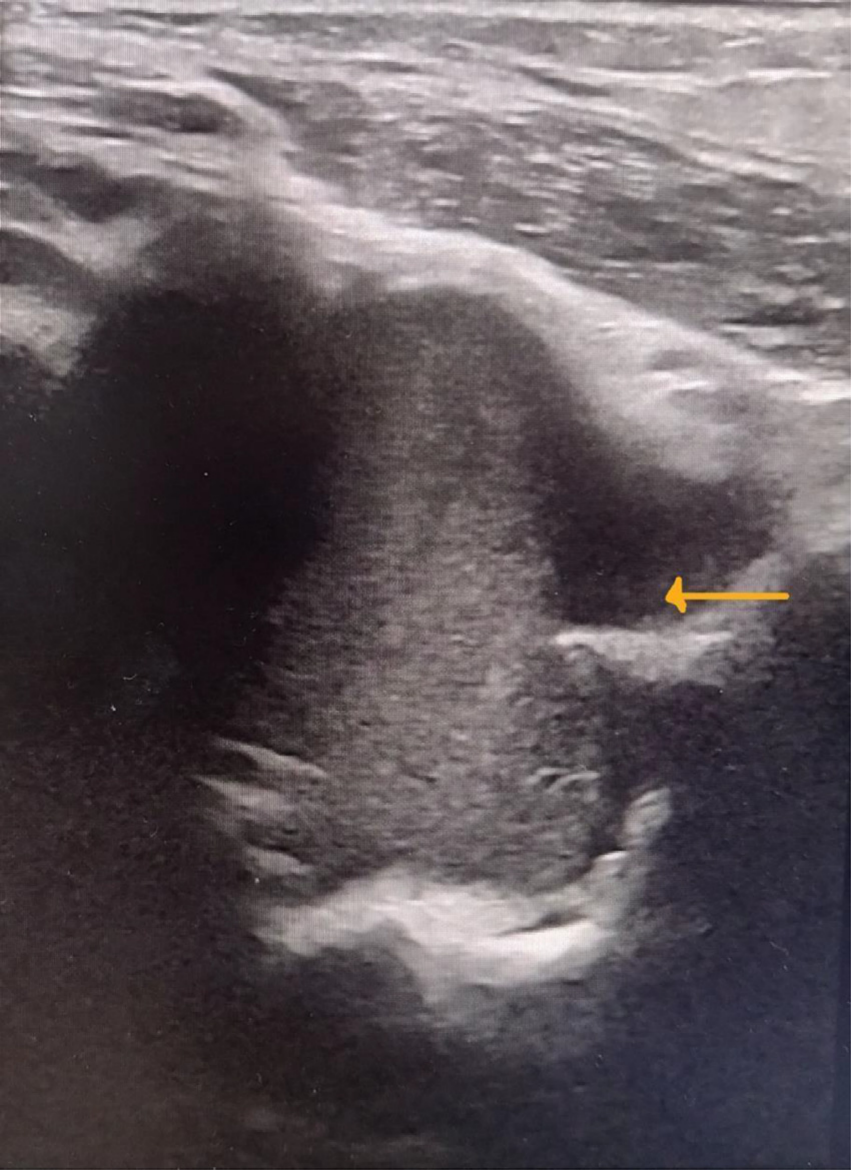
Ultrasound of left elbow joint. Yellow arrow points to joint effusion.

CECT brain, abdomen and pelvis failed to demonstrate any deep-seated collections or abscesses. Ultrasonic effusions were noted within both anterior and posterior recess of left elbow joint measuring 31 mm × 20 mm × 10 mm and 40 mm × 32 mm × 12 mm respectively (Fig. 2).

Ultrasound scan of the right ankle joint revealed tiny right joint effusion in the anterior joint recess measuring 4 mm in depth, expanded to retrocalcaneal bursa with fluid measuring 14 mm × 8 mm × 17 mm and an effusion adjacent to the 1st metacarpophalangeal joint measuring 13 mm × 4 mm. USS guided aspiration of the right ankle joint demonstrated 5000/mL polymorphs with negative bacterial cultures, suggestive of reactive arthritis.

USS guided aspiration was performed on left elbow joint which revealed 340000/mL polymorphs. Two peripheral blood cultures, sputum culture and left elbow joint aspirate culture grew the organism *Burkholderia psuedomallei. B. pseudomallei* that grew on the cultures, was sensitive to meropenem and trimethoprim/sulfamethoxazole and was intermediate susceptible to ceftazidime. He was extensively investigated for cellular or humoral immunodeficiency. His immunoglobulin levels and lymphocyte panel were normal with negative retroviral studies.

Under care of a multidisciplinary team which included a general physician, an infectious disease specialist and an orthopaedic surgeon he was managed as a case of disseminated melioidosis with septic arthritis. In addition to intravenous meropenem (1 g three timed a day), oral trimethoprim sulfamethoxazole (1920 mg twice a day) was also co-administered in the intensive phase. He underwent multiple attempts of joint washouts of left elbow joint, until the joint cultures became negative. He improved clinically and biochemically with antibiotics and joint draining. His right ankle joint effusion settled with analgesics. After 4 weeks of intensive phase treatment, he was switched to oral trimethoprim-sulfamethoxazole (1920 mg twice a day) eradication therapy for 3 months. He was monitored closely for recurrence of symptoms and cytopenic side effects of treatment. At the end of eradication therapy, our patient was symptoms free with normal exercise tolerance and normal joint mobility of right ankle and left elbow joints.

## 3 Discussion

*Burkholderia pseudomallei*, an environmental Gram-negative bacillus which can be found in damp soil and water, is the source of the infectious disease melioidosis, which can affect both humans and animals. Tropical regions, particularly those in Northern Australia and Southeast Asia, are the primary habitat for this disease. After a melioidosis epidemic in sheep in Central West Queensland in 1949, melioidosis was first discovered in Australia. However, the human cases were not recorded until 1950, when index cases were reported in Townsville, North Queensland. The annual incident rates of melioidosis differs across endemic Northern Australia and non-endemic South-west Australia, ranging from (5.4–50.2)/100 000 population and (2.7–10.0)/100 000 population respectively.^3^ Indigenous Australians are disproportionately impacted and bear the greatest burden of the disease, with a mean incidence of 33.1/100 000 population in Torres Strait Islanders.^3^ Prospective clinical studies from Northern Australia conducted in predominantly rural areas, found an increase in melioidosis cases after heavy rainfall or extreme weather events such as tropical storms or monsoons.^4^

In Australia, melioidosis is regarded as an opportunistic illness in humans, and the great majority of cases are associated with at least one risk factor. Diabetes mellitus, hazardous alcohol use, chronic lung disease, and chronic kidney disease are the traditional risk factors described in literature.^5^ Clinical spectrum of melioidosis can vary from a simple skin infection and pneumonia to severe septicaemia with multiorgan failure. Studies have identified pneumonia as the most common presentation of melioidosis in Australia.^6^ Genitourinary involvement with prostate abscesses formation up to 21% of males has been described in literature.^7^ Currie et al.^8^ described that neurological melioidosis, with brain and spinal cord involvement, can occur up to 5 % of Australian melioidosis cohort.

A 20-year prospective study conducted by Morse et al.,^9^ in Northern Australia involving 536 melioidosis patients, described that 2.4% of the cohort presented with primary septic arthritis, 1.3% with primary osteomyelitis and 3.9% with secondary septic arthritis or osteomyelitis with primary melioidosis elsewhere. The primary sites identified were pneumonia, bacteraemia with no focus, genitourinary disease and soft tissue abscess.^9^ Bone and joint involvement in melioidosis in Australian cohort was 7.6%, compared to 14%–27% reported in previous series of patients from Northeast Thailand.^10^ The disparity was ascribed to potential variations across the centres in the mean duration of receiving suitable therapy for melioidosis, wherein delays beyond two weeks are believed to provide a risk factor for bone and joint involvement.^11^ In addition, the Australian study described that, in the cohort for skeletal melioidosis, women were overrepresented, even though melioidosis is more frequent in men overall. In the Australian group, joint involvement was more common in the lower extremities, specifically the knee and ankle.^9^

Treatment of melioidosis in Australia consists of two phases; an intensive phase with intravenous antibiotics; meropenem or ceftazidime; followed by a prolonged eradication phase with oral antibiotics; trimethoprim-sulfamethoxazole, amoxicillin-clavulanate or doxycycline.^12^ Patients with neurological, bone or joint or genitourinary infections, in addition to intravenous antibiotics, oral trimethoprim-sulfamethoxazole is also co- administered in the intensive phase. Due to concerns regarding compliance to prolonged oral antibiotic therapy, in the endemic Northern Australia, a longer intravenous therapy is recommended during intensive phase.^13^

Several studies have emphasized the importance of having adjunctive open arthrotomy in managing joint infection in melioidosis.^14^ Chittrakarn et al.^15^ demonstrated that the in-hospital mortality and duration of hospital stays of patients who underwent adjunctive therapy with open arthrotomy were more favorable than those who did not. They also described that the patients who underwent adjunctive therapy with open arthrotomy had lower hospital expenditures than those who did not underwent an open arthrotomy.

The mortality from acute melioidosis is 20%–50% worldwide. Studies emphasize the importance of rapid disease recognition, appropriate antibiotics, adjunctive therapy (arthrotomy, abscess drainage) and access to intensive care as the outcome of the disease correlates with them and host co-morbidities.^16^

## 4 Conclusion

Our patient had septic arthritis with primary lung melioidosis, which is a relatively uncommon presentation for Australian population. The host factors; uncontrolled diabetes mellitus and ethanol misuse; and environmental factors; exposure to wet soil; increased his risk of contracting this opportunistic infection. His diagnosis of pulmonary melioidosis was delayed for 5 weeks, leading to a septicaemia and septic arthritis.

Many doctors in nonendemic areas find it difficult to diagnose melioidosis due to its broad spectrum of clinical presentations and protracted latency period. Prompt diagnosis would avert catastrophic outcomes.

Our case report highlights the importance of improved clinical awareness among health practitioners and a low threshold for radiological screening of patients with risk factors for melioidosis, even in non-endemic areas. It also highlights the fact that performing adjunctive open arthrotomy in managing joint infection in melioidosis, in addition to antibiotics treatment, improves the clinical response to treatment.

## Funding

None.

## CRediT authorship contribution statement

**Buddhika Dhananjalee Alahakoon:** Writing – review & editing, Writing – original draft, Visualization, Validation, Supervision, Software, Resources, Project administration, Methodology, Investigation, Funding acquisition, Formal analysis, Data curation, Conceptualization. **Monarita Handa:** Writing – original draft, Formal analysis, Data curation, Conceptualization. **Shiromali Malalasekara:** Writing – review & editing, Writing – original draft, Validation, Supervision, Project administration, Investigation, Formal analysis, Data curation, Conceptualization.

## Appendix Supplementary materials

Image, application 1
