# Supplementary figures and images for: A case report from non-endemic Australia on systemic melioidosis presenting with septic arthritis
Source: Infect Med (Beijing). 2024 Dec 31;4(1):100161. doi: 10.1016/j.imj.2024.100161 (PMC11795047; doi:10.1016/j.imj.2024.100161)

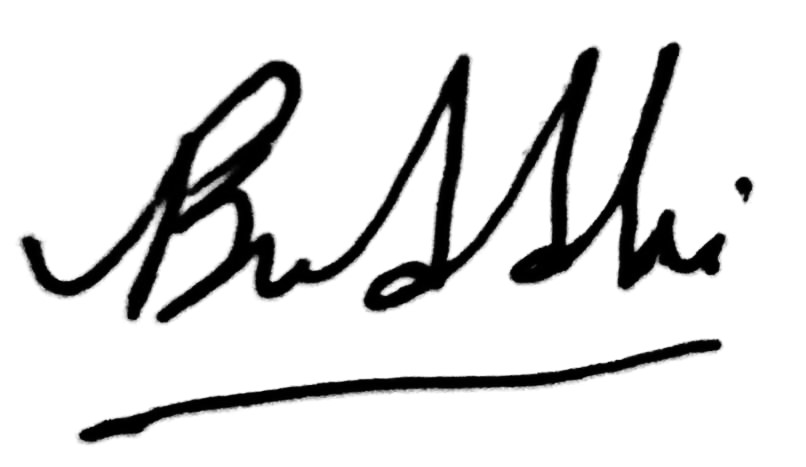

Supplement: Supplementary file 1 [file mmc1.zip › image.jpg]
